# Supplementary material for: Sequence-dependent material properties of biomolecular condensates and their relation to dilute phase conformations
Source: Nat Commun. 2024 Mar 1;15:1912. doi: 10.1038/s41467-024-46223-w (PMC10907393; doi:10.1038/s41467-024-46223-w)
Supplement: Supplementary file 1 — Supplementary Information [file 41467_2024_46223_MOESM1_ESM.pdf]

Supplementary Information

Sequence-Dependent Material Properties of Biomolecular Condensates and their Relation to Dilute Phase Conformations

Dinesh Sundaravadivelu Devarajan,[1,*] Jiahui Wang,[1] Beata Szała-Mendyk,[1] Shiv Rekhi,[1] Arash Nikoubashman,[2,3,4] Young C. Kim,[5] and Jeetain Mittal[1,6,7,*]

[1]Artie McFerrin Department of Chemical Engineering, Texas A&M University, College Station, TX 77843, United States

[2]Leibniz-Institut für Polymerforschung Dresden e.V., Hohe Straße 6, 01069 Dresden, Germany

[3]Institut für Theoretische Physik, Technische Universität Dresden, 01069 Dresden , Germany

[4]Cluster of Excellence Physics of Life, Technische Universität Dresden, 01062 Dresden, Germany

[5]Center for Materials Physics and Technology, Naval Research Laboratory, Washington, DC 20375, United States

[6]Department of Chemistry, Texas A&M University, College Station, TX 77843, United States

[7]Interdisciplinary Graduate Program in Genetics and Genomics, Texas A&M University, College Station, TX 77843, United States

[*]Corresponding authors' email: dineshsd@tamu.edu, jeetain@tamu.edu

Supplementary Table 1. Amino acid sequences of E−K, LAF1, and DDX4 protein variants that were used in this study.

**E−K**	**Sequence**
nSCD = 0.000	EKEKEKEKEKEKEKEKEKEKEKEKEKEKEKEKEKEKEKEKEKEKEK<br>EKEK
nSCD = 0.017	KEKKKEKEEEKKEKEKKEEKEKEEKKEEKKEEEKKKEEEKKEKKKE<br>EEEK
nSCD = 0.033	KEKKKEKEEEKEEEKKEEKEEEKEEKKKKKEEEKKKEKEEEEKEKKK<br>EKEK
nSCD = 0.059	KKEEKEKEEEKEEEEEEKKKKEEKEKEKKKEKKEEEKKKEEKKKKE<br>EEKK
nSCD = 0.067	KKEEKEKKEEEEEKEEEKEEKKKEEEEKKKKKKKKEKKEKEEEEKEK<br>KEEK
nSCD = 0.200	EEKKEKEEEEEEKEKEEEEEEEKEEKEKKKKKKKKEKEKKKEKKKEK<br>EEKK
nSCD = 0.333	EEEKEKEKEKEEEEEEEEEEEKEEKEKEKKKKKKEEKKKEKKKKKE<br>KKKK
nSCD = 0.400	EKEKEEEEEEKEEEEEEEEKEEEKKEKEKKKKKKEEEKEKKKKKKK<br>KKKK
nSCD = 0.468	EEEEEEEEEKEEEEEKEEKKKEKKEKEEEKKKEEKEKKKKKKKK<br>KKKK
nSCD = 0.499	EEEEEEEEEKEEEEEEEEEKKKEKKKKEEEKKKEKKEKKEKKKKK<br>KKKK
nSCD = 0.601	EEEEEEEEEEEEEEEEKEEKKEKEEKKEKKKEKEKKKKEKKKKKK<br>KKKK
nSCD = 0.667	EEEEEEEEEEEEEEEEKEEEKEKEKEKEKKKKKKKKEKKKKKKKKE<br>KKKK
nSCD = 0.734	EEEEEEEEEEEEEEEKEEEEEKKEEEEKKKKKKKKKEKKKKKKK<br>KKKK
nSCD = 0.902	EEEEEEEEEEEEEEEEEEEEEKEKEKEKEKKKKKKKKKKKKKKKK<br>KKKK

nSCD = 1.000	EEEEEEEEEEEEEEEEEEEEEEEEEKKKKKKKKKKKKKKKKKKKKKKKKK
**LAF1**	**Sequence**
nSCD = 0.003	MGGSGGRGSGRNGDYYGSGPGYRAQDYGHGDSRRGGANSSGGNGGNPGRAEGREGGGGANGYYSGGGANQASGGNRNGGADGGGGGDRGGGNNGNRGDAGYREGNRRGDNNGNFGDNNSDRGLRGASDRDGNRNYLGSRDRDVNGGGDYNYRNGGYRNRGDDGRSGDR
nSCD = 0.010 **Wild-type**	MESNQSNNGGSGNAALNRGGRYVPPHLRGGDGGAAAAASAGGDDRRGGAGGGGYRRGGGNSGGGGGGGYDRGYNDNRDDRDNRGGSGGYGRDRNYEDRGYNGGGGGGGNRGYNNNRGGGGGGYNRQDRGDGGSSNFSRGGYNNRDEGSDNRGSGRSYNNDRRDNGGDG
nSCD = 0.039	MRRGGYGGDGGLGNGHNDERRNARRGQNNSGFGYDAGRRSRGRYGAGGGDRNGRNDSNGLGGNSGDNGGGRNGANRGYRGDDNNARGSNVGRYSDDGARREGSSSGGGGYGNNGGGNGDGPGANGRDAYRGNDGGPYQRSGYAGSNGGRDGDNSDYYDGGNRGEGGGG
nSCD = 0.131	MGGDRAGYNNGDRLNRNGFRRLSGPNGAGGGNSDRRYYRNARRGGGRGGRAGGYGYNGGRRRAQGSRGEGGGGGGSYDNGNRGEGSGRGRNNDGGGDDNARNNDSHRDPGRVGNAGYGGGGGNGNGSYYGGDNNDAGGSGSADGGGSNGDYDERGDSNGRDGNGQYDS
nSCD = 0.206	MNNSGDNDRGSGNYGLRNSFGDDGYGDNGNDEGNSGYRNRGLGGDRADEYGNSGGNGDNEAAPNASDRDDAHYYDSDDYDDGGGGRGSGGAGGGGARGPGSNRAGRYGGGGRRGRGRGNGYNGNRSQRRRGGGRGRGNRGYRVGNGNGQSGGRNSRGGGGGNGGANYG
nSCD = 0.263	MDGGDDDGGQGGAGENLNDYYNGSSGGPGNSVAENSGGNGNDDGGGDNRNNRYYSGGGDGRYGGGGQGANGGDDNAGDGYDGDNFGNGNGRGYNNGSYGADASEGDRGSYRSSGRRRRRGSGGGGNYPGGGSRRLNRNRGARGGAGGNRRRNARRDGNGYGGRRGRGH
nSCD = 0.394	MESNQSNNGGSGNAALNDGGDYVPPHLDGGDGGAAAAASAGGDEDDGGAGGGGYDDGGGNSGGGGGGGYDEGYNDNDDDDNDGGSGGYGDRRNYRRRGYNGGGGGGGNRGYNNNRGGGGGGYNRQRRGRGGSSNFSRGGYNNRRRGSRNRGSGRSYNNRRRRNGGRGR

DDX4	Sequence
nSCD = 0.002	MDPTESFCANGGGAGSNKMGDGGNRATSDGLKNVFSMSNEADGRS<br>SDRSGNSNSGDPKDKGGETSRESRNGSGGFERGREPGNSGRNIGSSTI<br>EERLSRISGSDGTPYLDGSYSRERVSADHGGNMFYCFPSDNKGGSGL<br>RPNANEFNRERTSQVMDRPNGEGFFRSFYRHPVGGMESTYWGQSGS<br>GPERNGFGSDDGWRLSFRKDEWRCSDKDNGFNEFRKSGNGGERCT<br>GEGDF
nSCD = 0.010	MGRDRLRGDNDLPKGYEGFENEHFSKSNTKGTRGPVNNEGDRGFN<br>DGSYNGIGSRGQFGDGSCEPGGNMKMGVTGGGCSISEEGPSEENNR<br>RNRWRQFGNDSGDGWVPGPACHRGISGFFRKDTSGKSDCDERTSAR<br>SREEGDSGLDSSYWGEGSFRSASRRRNRGDSNELDSGSEFYGGNENP<br>GMRSDAFTGSGEPFSPDSSKMNFTSSAYFFANDVKELGSNTSRGRNG<br>GGRMS
nSCD = 0.021<br>**Wild-type**	MGDEDWEAEINPHMSSYVPIFEKDRYSGENGDNFNRTPASSSEMDD<br>GPSRRDHFMKSGFASGRNFGNRDAGECNKRDNTSTMGGFGVGKSF<br>GNRGFSNSRFEDGDSSGFWRESSNDCEDNPTRNRGFSKRGGYRDGN<br>NSEASGPYRRGGRGSFRGCRGGFGLGSPNNDLDPDECMQRTGGLFG<br>SRRPVLSGTGNGDTSQSRSGSGSERGGYKGLNEEVITGSGKNSWKSE<br>AEGGES
nSCD = 0.095	MGDPDDDEEEKGSNSTGGEQDGELGQDVSANDLGGSRSTSEDEFSR<br>EYNEGPSYGYSTGRFKDWRFTEFSFREFTFMNGPGNKGEIDGRWHIF<br>DNAGCSDSGPRGDITESGGSSSSNNKRAGGVKNRNDGEGDSMPGGR<br>GTRNGRHRGGSPNRRPFVAGSNSDRNNLSCNGSLEWYGGEGPCRSP<br>RMGGGAGNMSGGYRKGDERMSLVNGATGKFNRDERRRSFSSCFFN<br>SSFKSS
nSCD = 0.194	MRNESEGEYCCEADDFVGNPSDNSYTRASTGSDDDRGFYFSFEAGG<br>SYDEFSSEGDEWDNNGSGGGDNFNESDFRGDNETGDPERGDNDGIE<br>KHGDPGISPLGRSPGMSRTTDTRGSMLGGSFEVGSGFLFTGESGSAS<br>MGWRGNSRFKFCRDMLQASKRINNNKVSGGRRSGESWCSGGREGT<br>GSSMRGRGPKFEGNRGRSPNNKAQGKRRRPFGGKHNRYNSERNGSL<br>NVSSGP

nSCD = 0.284	MNSEEYCGCEADDDFVGNPSNSYTRASTGSRDDEDGFFSYDEEEEEE EFSSGDWDNNGSGGGEEDNFNSDFIKHRGDNETGDGSPGFGNGYFS GDDPGISPLGSPGMSERRRRTTDDDTGGGSFVAGGLFTGSGSASMG WSMFKRRFLCRRRKMLQASGNSINVSSNNKVSGGSGESWCSGGRRG TGSSMKGGPFEGNGSPNNKAQGKRRRRRPFGGKHNRRRRRYNSEN GSLNGP
nSCD = 0.478	MNESEEGYCACEDDEFVGNPSDNSEEYTASTGSDDDEEEEEEDEGFY FSFAGGSYFSSGDWDNNGSGGGDNFNSDFGDNETGDPEGDNDGIEK HGDDPGISPLGSPGMSRTTTRRGSMLGGSFEVGSGFLFTGSGSASMG WRRRRGNSFKFGSWCSCDMLQASINNVGGNKNKVSSLRRRRRSGG GTGSSMGKRRGPFGGNSPNNKAQGRRRRRRRRRPFGGKHNYNSRR NGSSGP

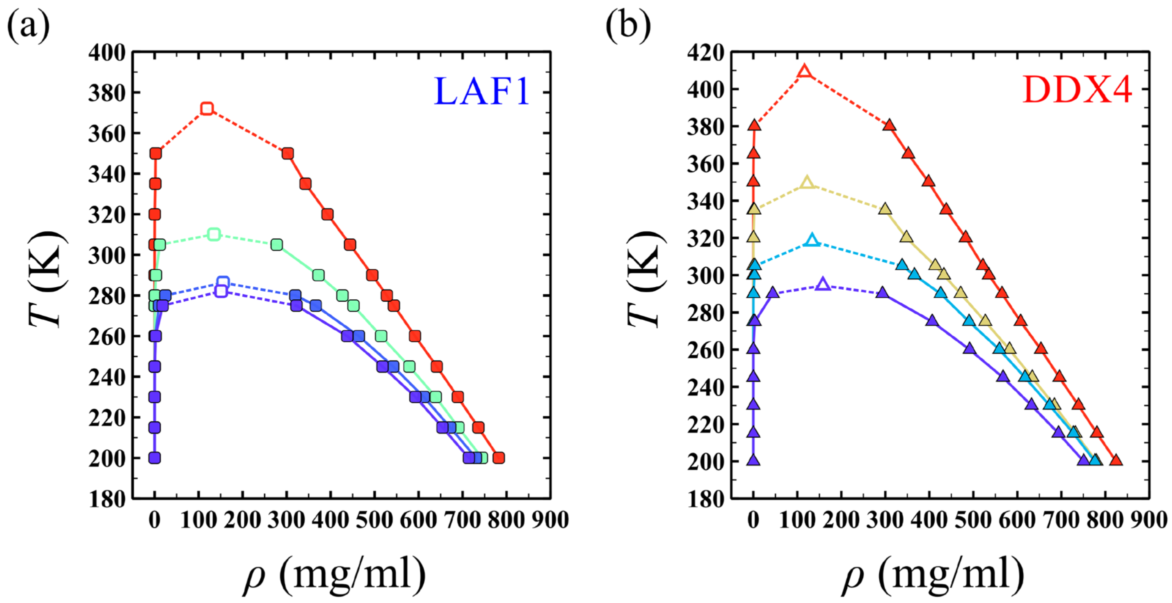

Supplementary Fig. 1. Phase diagrams for select sequence variants of (a) LAF1 (nSCD = 0.003, 0.010, 0.131, and 0.394) and (b) DDX4 (nSCD = 0.002, 0.021, 0.194, and 0.478). The open symbols correspond to the critical temperature and critical density of the sequence variants. The line color, ranging from blue to red, indicates increasing nSCD. Lines are guide to the eye only. Source data are provided as a Source Data file.

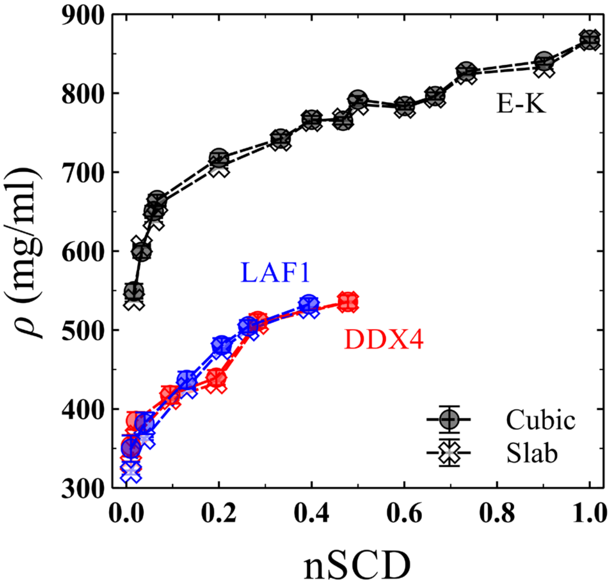

Supplementary Fig. 2. Comparison between the dense phase concentration ρ attained by the E−K, LAF1, and DDX4 sequence variants in cubic and slab geometries. Error bars indicate standard deviations about the mean. The mean values are obtained as an average over $n = 2$ independent simulations, with each replica divided into 3 independent blocks to estimate error bars. Source data are provided as a Source Data file.

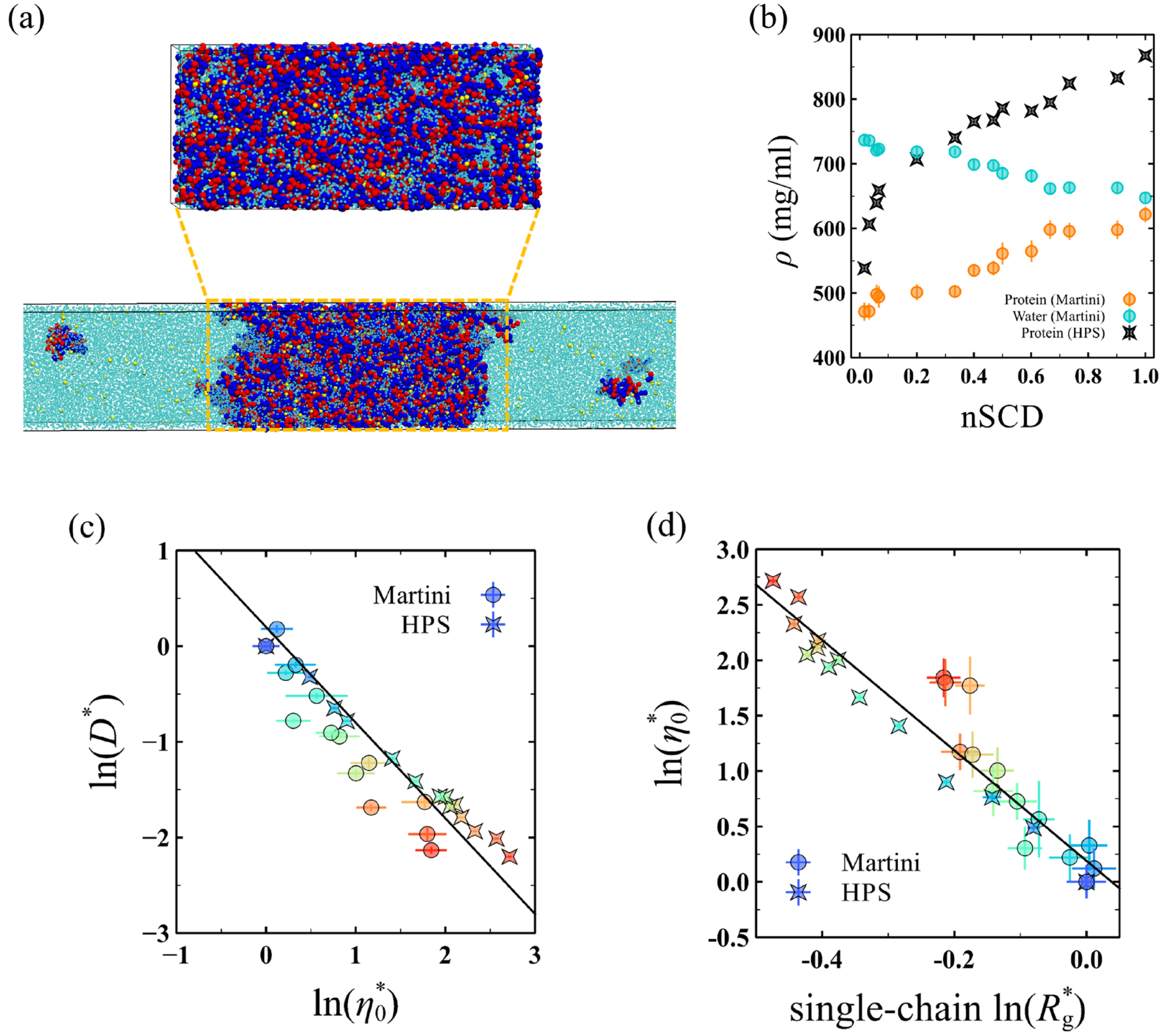

Supplementary Fig. 3. (a) Dense phase snapshot of Martini set-up for one of the E−K sequences in the presence of water (cyan) and ions (yellow) in a cuboid simulation box, which was cut from a slab geometry, for characterizing its dense phase diffusive and rheological properties. (b) Comparison of the dense phase concentration ρ for protein and water as a function of nSCD in the Martini model with the protein's concentration in the HPS model for the E−K sequences. (c) Inversely proportional correlation between normalized diffusion coefficient D^* and normalized zero-shear viscosity η_0^* from the Martini and HPS models for the E−K sequences, with the solid line corresponding to the Stokes-Einstein type relation $D^* = 1/\eta_0^*$. (d) Correlation between η_0^* and normalized single-chain R_g^* from the Martini and HPS models for the E−K sequences. The solid line corresponds to the linear fit $\ln(\eta_0^*) = -4.98\ln(R_g^*) + 0.19$ with correlation coefficient $R^2 = 0.90$. The values of D^*, η_0^*, and R_g^* are obtained after normalizing D, η_0, and R_g by those computed for the reference E−K sequence with nSCD $= 0.017$ in the HPS and Martini models. The symbol color, ranging from blue to red in (c) and (d), indicates increasing nSCD. Error bars in (b), (c), and (d) indicate standard errors about the mean.

The mean values are obtained as an average over $n = 2$ independent simulations for ρ and D^*, over $n = 5$ independent simulations for R_{g}^*, and over $n = 10$ independent simulations for η_0^*, with each replica divided into 3 independent blocks to estimate error bars. Source data are provided as a Source Data file.

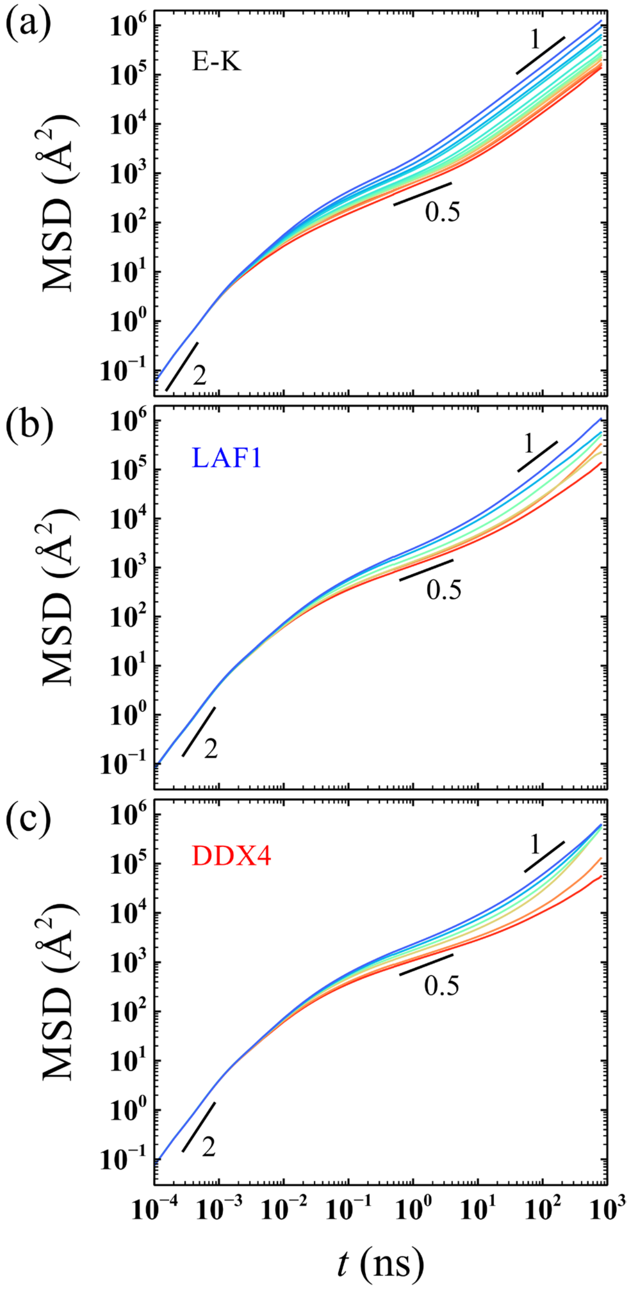

Supplementary Fig. 4. Mean square displacement MSD(t) of the residues of a chain in the dense phase for the (a) E−K, (b) LAF1, and (c) DDX4 sequences. To avoid chain end effects, 20 residues on either end of a chain have not been considered for computing the MSD. The slope values 2, 0.5, and 1 indicate ballistic, sub-diffusive, and diffusive regimes, respectively. The line color, ranging from blue to red, indicates increasing nSCD. Source data are provided as a Source Data file.

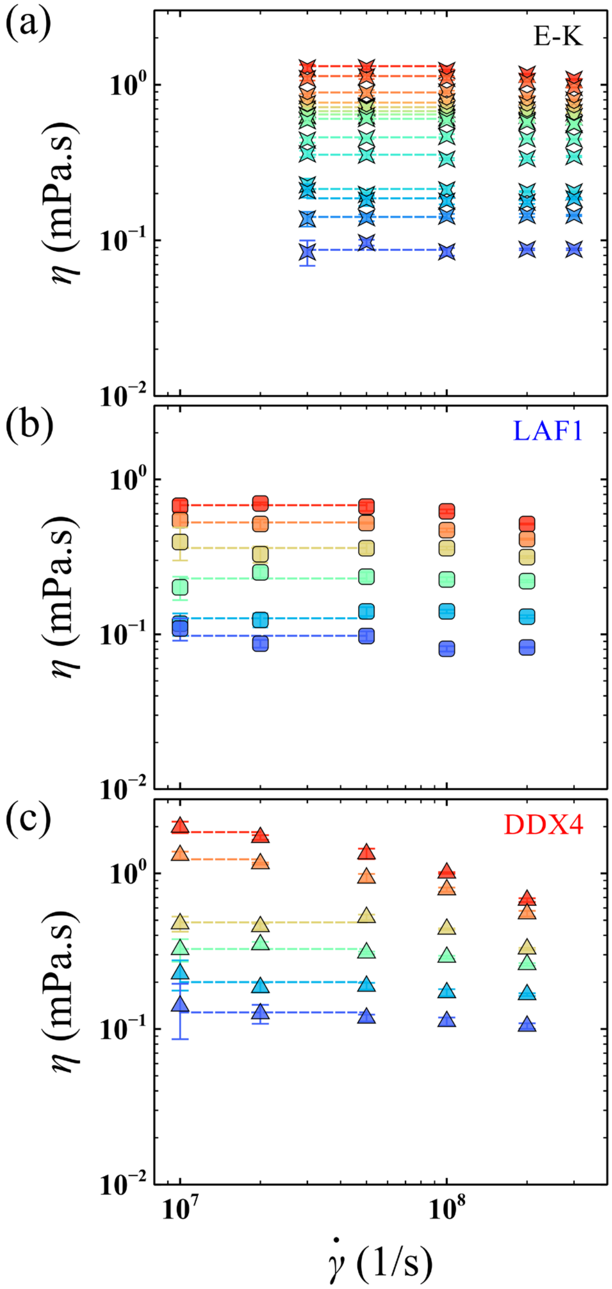

Supplementary Fig. 5. Shear viscosity η as a function of shear rate $\dot{\gamma}$ for the (a) E−K, (b) LAF1, and (c) DDX4 sequences in the dense phase. The dashed lines indicate the zero-shear viscosity η_0 of each sequence, which is obtained as an average of the near-constant η values from the Newtonian plateau observed at low shear rates. The symbol and line colors, ranging from blue to red, indicate increasing nSCD. Error bars in all panels indicate standard errors about the mean. The mean values are obtained as an average over $n = 2$ independent simulations, with each replica divided into 3 independent blocks to estimate error bars. Source data are provided as a Source Data file.

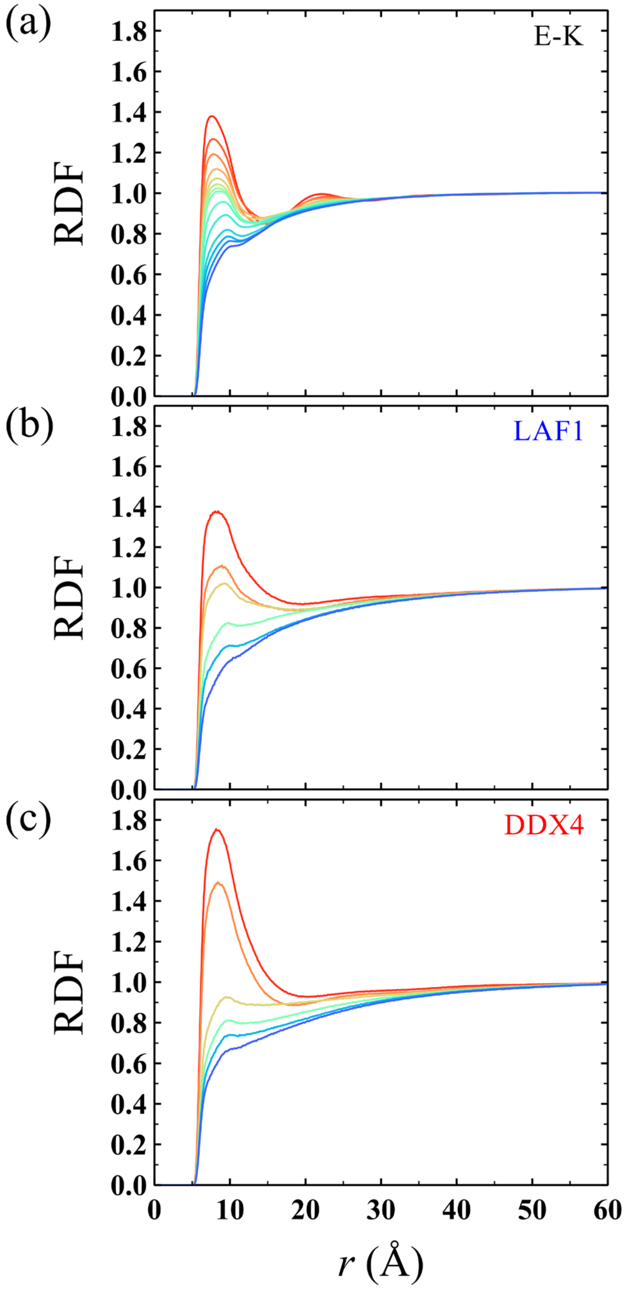

Supplementary Fig. 6. Radial distribution function (RDF) as a function of distance r between the interchain oppositely charged residues for the (a) E−K, (b) LAF1, and (c) DDX4 sequences in the dense phase. The line color, ranging from blue to red, indicates increasing nSCD. Source data are provided as a Source Data file.

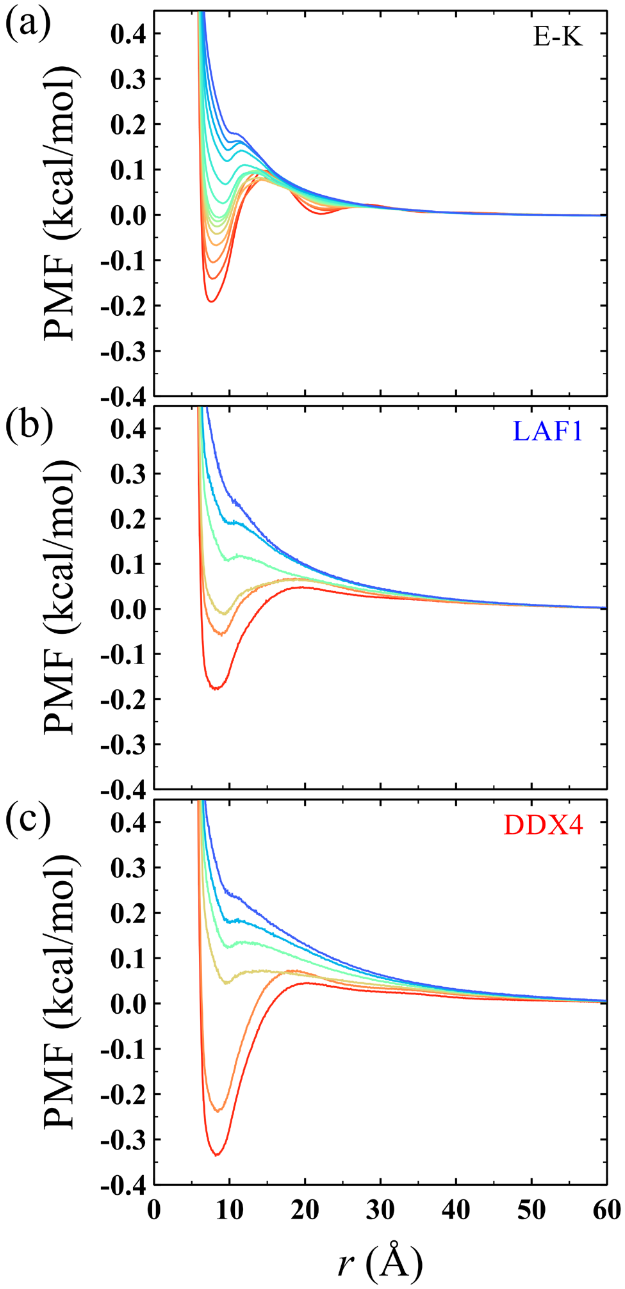

Supplementary Fig. 7. Potential of mean force (PMF) as a function of distance r between the interchain oppositely charged residues for the (a) E−K, (b) LAF1, and (c) DDX4 sequences in the dense phase. The line color, ranging from blue to red, indicates increasing nSCD. Source data are provided as a Source Data file.

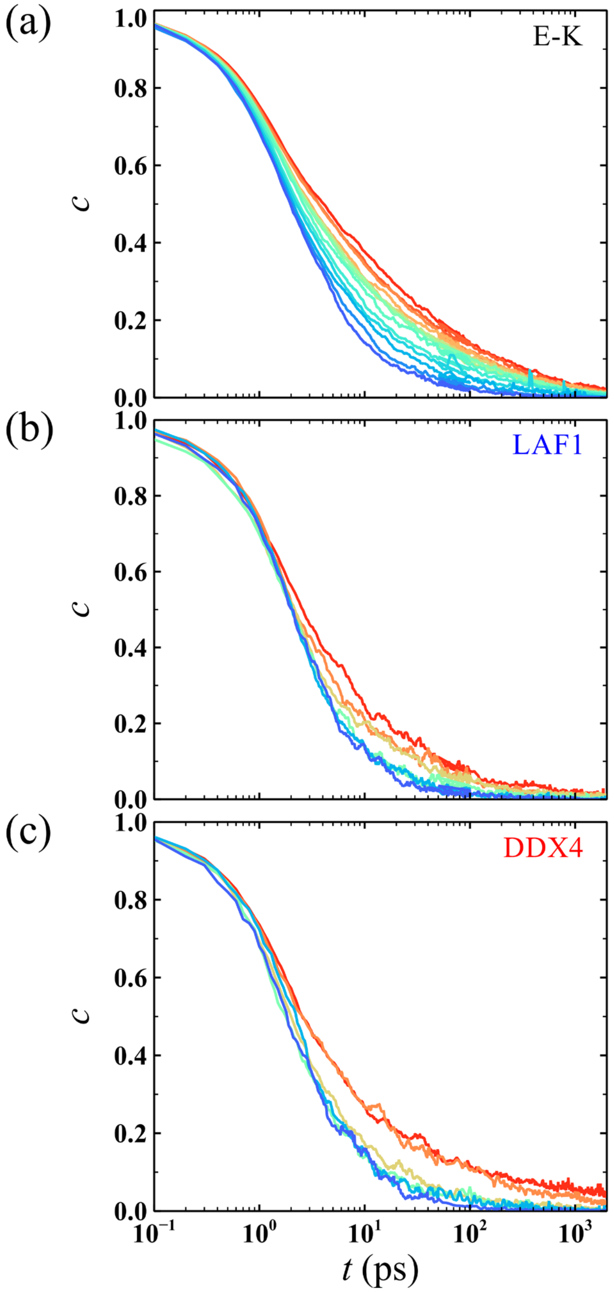

Supplementary Fig. 8. Intermittent contact time autocorrelation $c(t)$ between the interchain oppositely charged residues for the (a) E−K, (b) LAF1, and (c) DDX4 sequences in the dense phase. The line color, ranging from blue to red, indicates increasing nSCD. Source data are provided as a Source Data file.

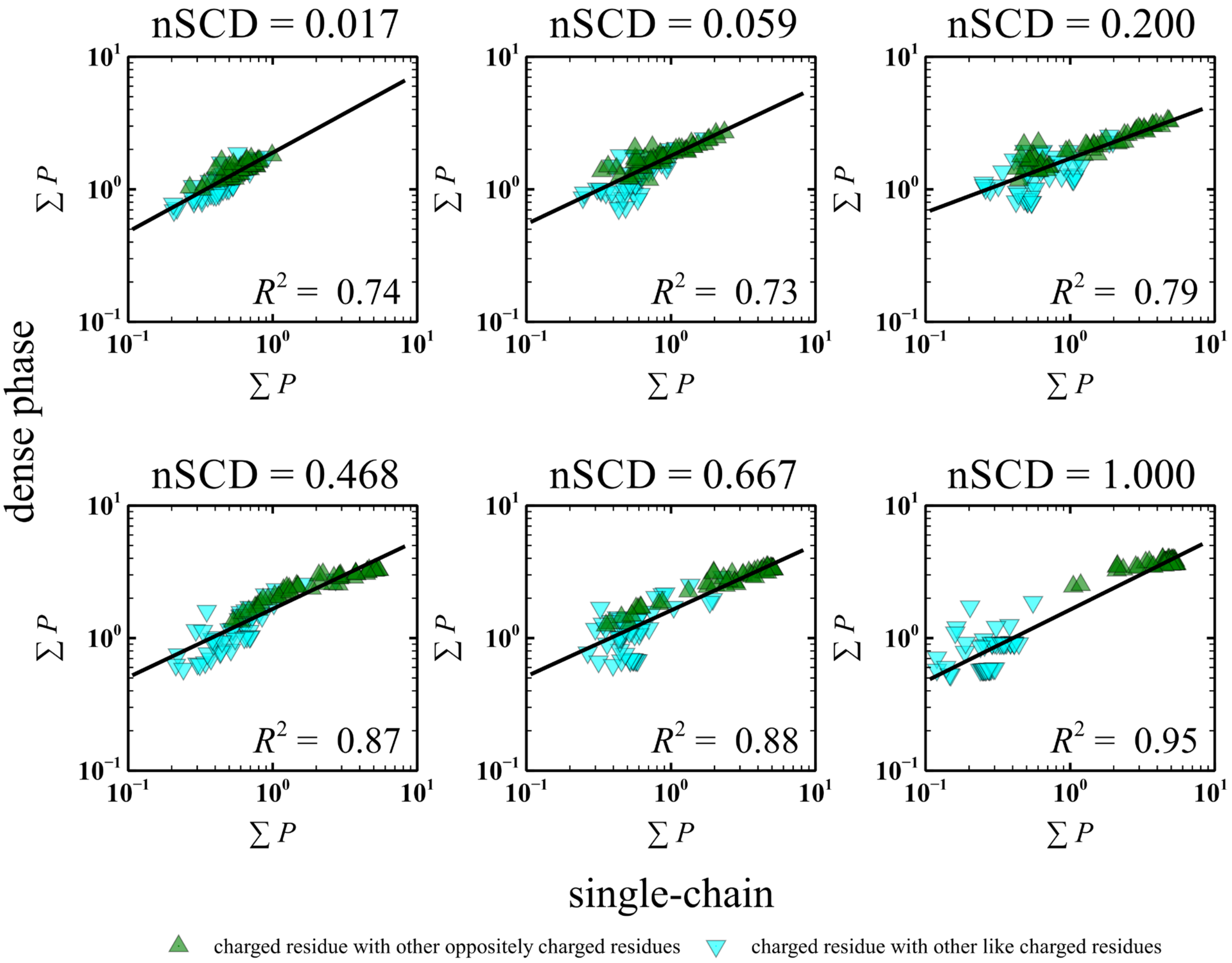

Supplementary Fig. 9. Correlation between the summed-up contact probabilities $\sum P$ of a residue i with all other oppositely charged residues or like charged residues for the chains in the dense phase and for a single-chain of select E−K sequences. For the single-chain $\sum P$, contributions from directly bonded residues and residues that are separated by two or three bonds are excluded as their contacts are primarily established by the chain connectivity. Source data are provided as a Source Data file.

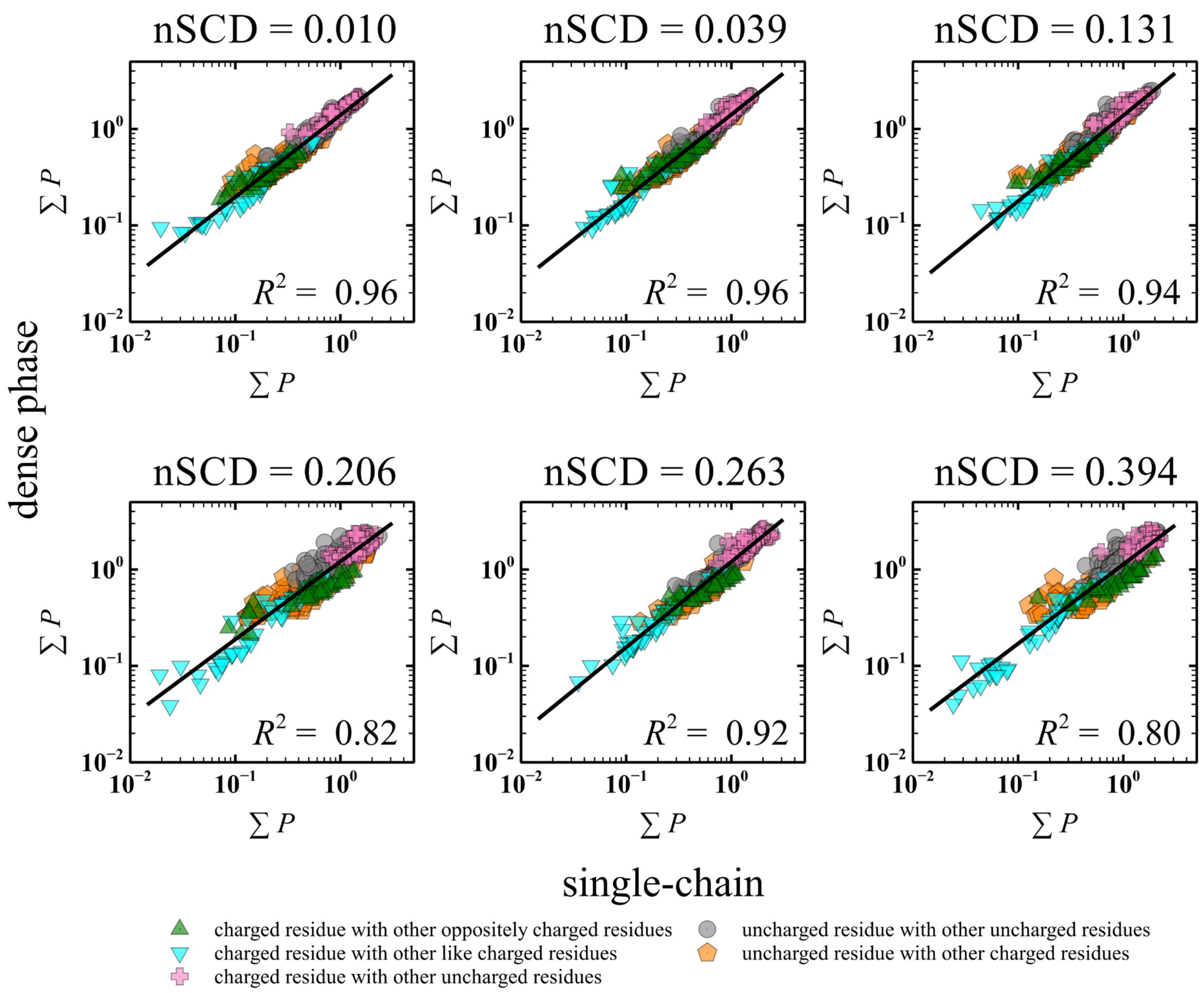

Supplementary Fig. 10. Correlation between the summed-up contact probabilities $\sum P$ of a residue i with all other oppositely charged residues or like charged residues or uncharged residues for the chains in the dense phase and for a single-chain of select LAF1 sequences. For the single-chain $\sum P$, contributions from directly bonded residues and residues that are separated by two or three bonds are excluded as their contacts are primarily established by the chain connectivity. Source data are provided as a Source Data file.

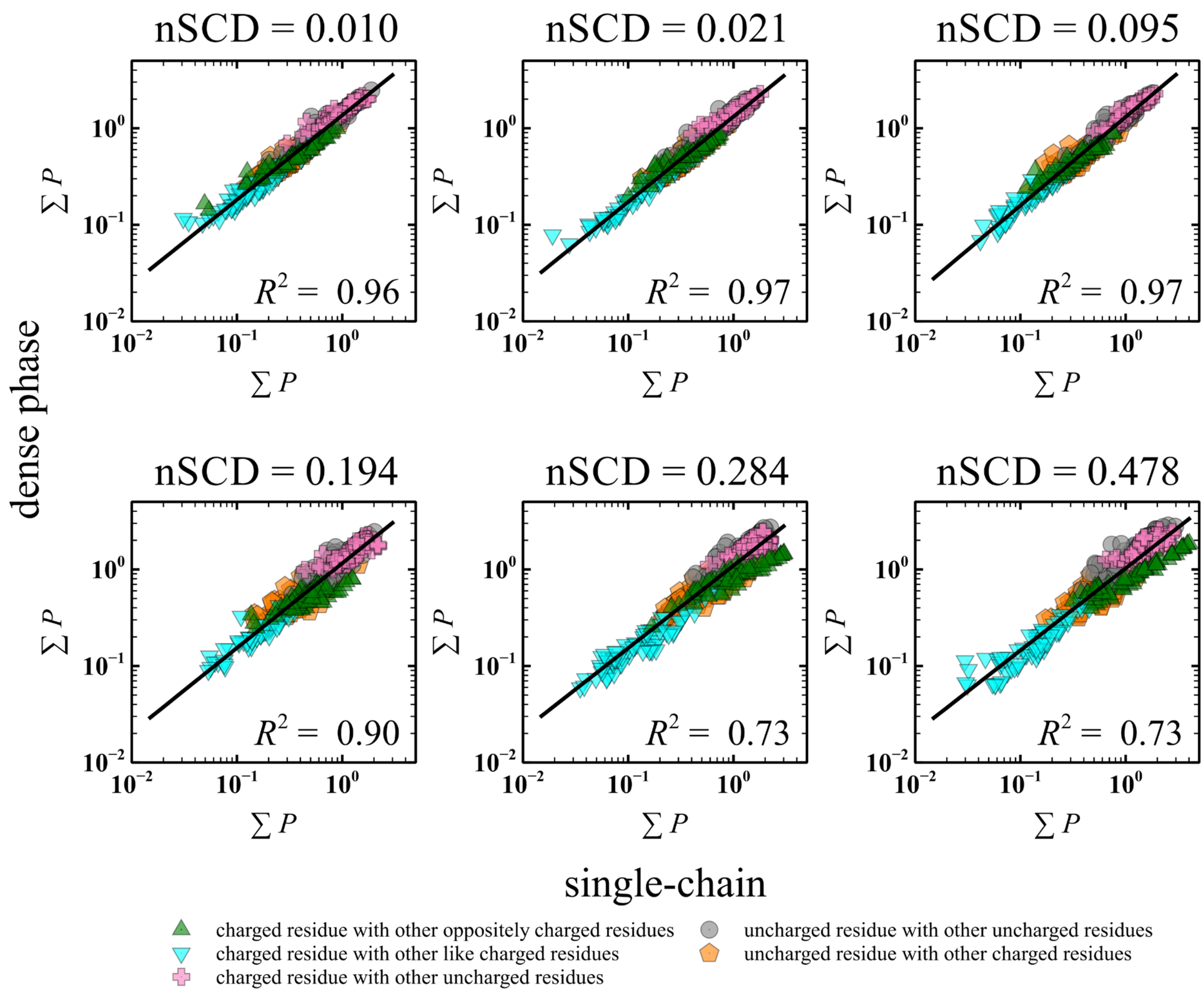

Supplementary Fig. 11. Correlation between the summed-up contact probabilities $\sum P$ of a residue i with all other oppositely charged residues or like charged residues or uncharged residues for the chains in the dense phase and for a single-chain of select DDX4 sequences. For the single-chain $\sum P$, contributions from directly bonded residues and residues that are separated by two or three bonds are excluded as their contacts are primarily established by the chain connectivity. Source data are provided as a Source Data file.

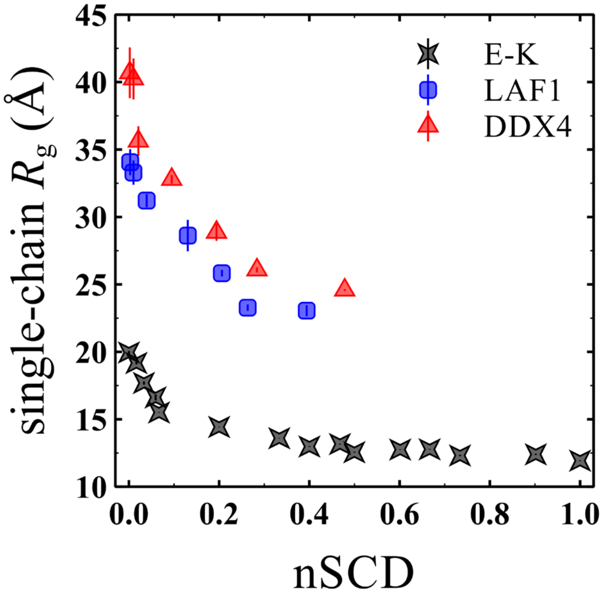

Supplementary Fig. 12. Radius of gyration R_g as a function of nSCD for the single-chain E−K, LAF1, and DDX4 sequences. Error bars indicate standard errors about the mean. The mean values are obtained as an average over $n = 2$ independent simulations, with each replica divided into 3 independent blocks to estimate error bars. Source data are provided as a Source Data file.

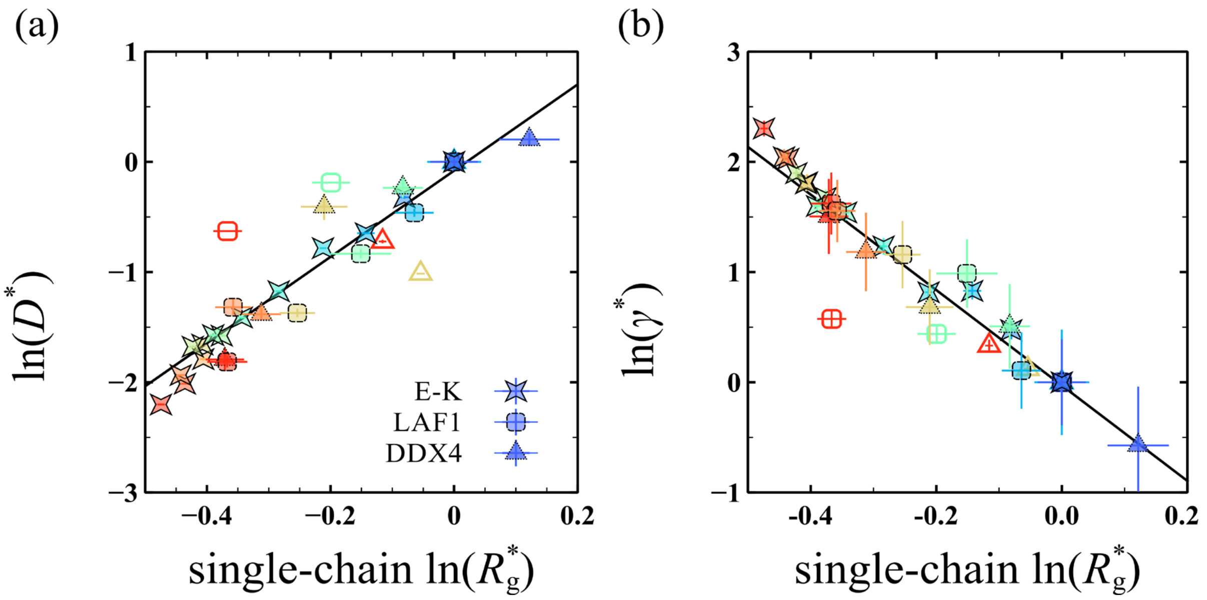

Supplementary Fig. 13. Correlation (a) between diffusivity D^* and single-chain R_g^*, and (b) between surface tension γ^* and single-chain R_g^* for all sequences. The symbol color, ranging from blue to red, indicates increasing nSCD. The values of D^*, γ^*, and R_g^* are obtained after normalizing D, γ, and R_g by those computed for the same reference sequences used in **Figs. 2 and 3**. The closed and open symbols correspond to the values obtained based on Urry's hydropathy scale and Kapcha-Rossky's hydropathy scale, respectively. The solid lines correspond to the linear fits $\ln(D^*) = 3.91\ln(R_g^*) - 0.08$ with correlation coefficient $R^2 = 0.84$, and $\ln(\gamma^*) = -4.33\ln(R_g^*) - 0.03$ with $R^2 = 0.91$, respectively. Error bars in all panels indicate standard errors about the mean. The mean values are obtained as an average over $n = 2$ independent simulations, with each replica divided into 3 independent blocks to estimate error bars. Source data are provided as a Source Data file.

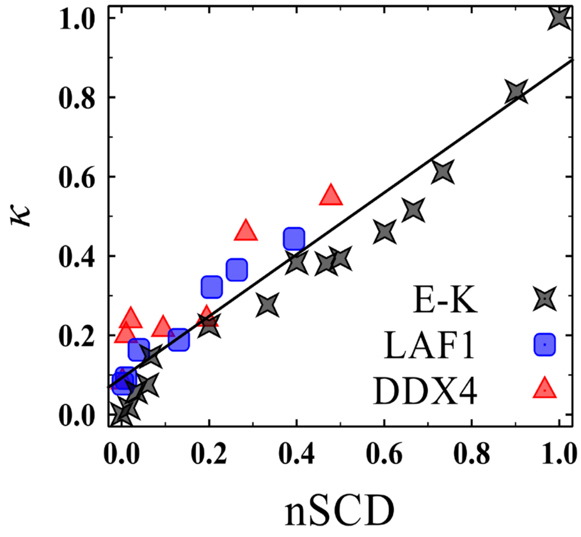

Supplementary Fig. 14. Correlation between nSCD parameter used in this work and κ parameter introduced by Das and Pappu.[2] The solid line corresponds to a linear fit with correlation coefficient $R^2 = 0.90$. Source data are provided as a Source Data file.

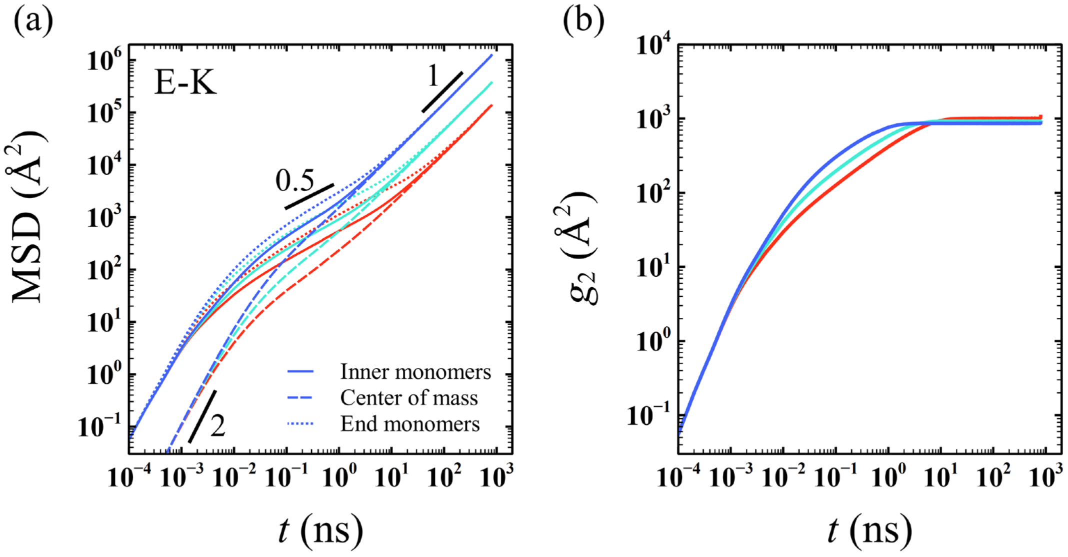

Supplementary Fig. 15. (a) Mean square displacement MSD(t) of the inner residues of a chain (solid lines), the center of mass of a chain (dashed lines), and the end monomers of a chain (dotted lines) in the dense phase for select E−K sequences (nSCD = 0.017, 0.200, and 1.000). The slope values 2, 0.5, and 1 indicate ballistic, sub-diffusive, and diffusive regimes, respectively. Inner residues correspond to those in the middle of a chain after excluding 20 residues on either end of the sequence. (b) MSD of the inner residues of a chain relative to the center of mass $g_2(t)$ in the dense phase for select E−K sequences. The line color, ranging from blue to red, indicates increasing nSCD. Source data are provided as a Source Data file.

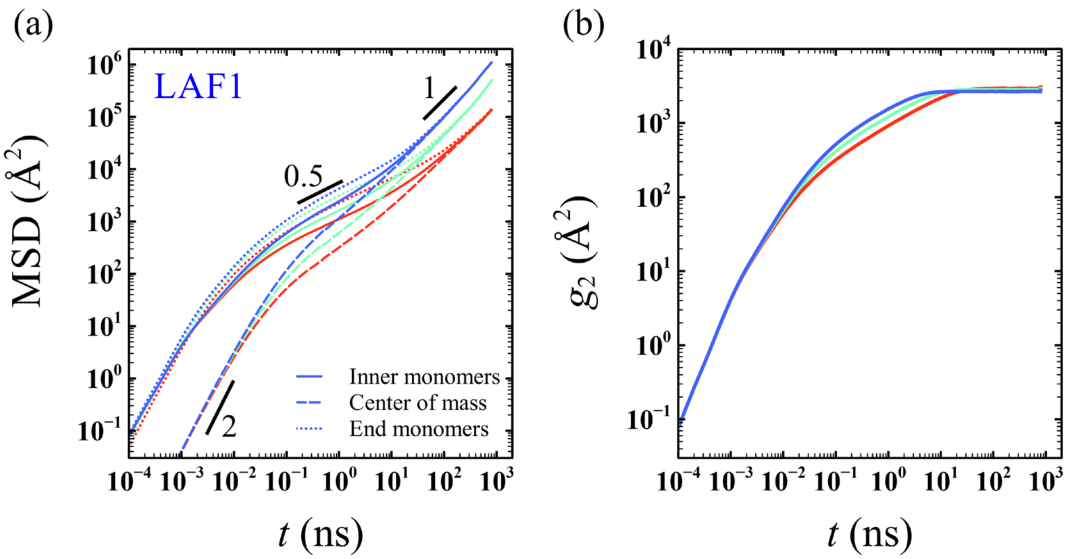

Supplementary Fig. 16. (a) Mean square displacement MSD(t) of the inner residues of a chain (solid lines), the center of mass of a chain (dashed lines), and the end monomers of a chain (dotted lines) in the dense phase for select LAF1 sequences (nSCD $= 0.010, 0.131,$ and 0.394). The slope values 2, 0.5, and 1 indicate ballistic, sub-diffusive, and diffusive regimes, respectively. Inner residues correspond to those in the middle of a chain after excluding 20 residues on either end of the sequence. (b) MSD of the inner residues of a chain relative to the center of mass $g_2(t)$ in the dense phase for select E−K sequences. The line color, ranging from blue to red, indicates increasing nSCD. Source data are provided as a Source Data file.

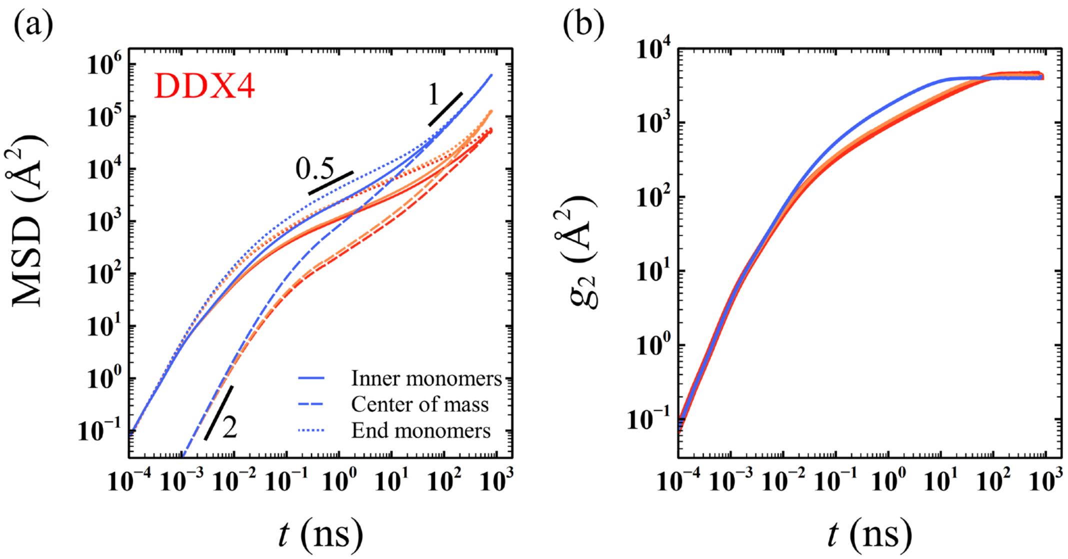

Supplementary Fig. 17. (a) Mean square displacement MSD(t) of the inner residues of a chain (solid lines), the center of mass of a chain (dashed lines), and the end monomers of a chain (dotted lines) in the dense phase for select DDX4 sequences (nSCD = 0.010, 0.284, and 0.478). The slope values 2, 0.5, and 1 indicate ballistic, sub-diffusive, and diffusive regimes, respectively. Inner residues correspond to those in the middle of a chain after excluding 20 residues on either end of the sequence. (b) MSD of the inner residues of a chain relative to the center of mass $g_2(t)$ in the dense phase for select E−K sequences. The line color, ranging from blue to red, indicates increasing nSCD. Source data are provided as a Source Data file.

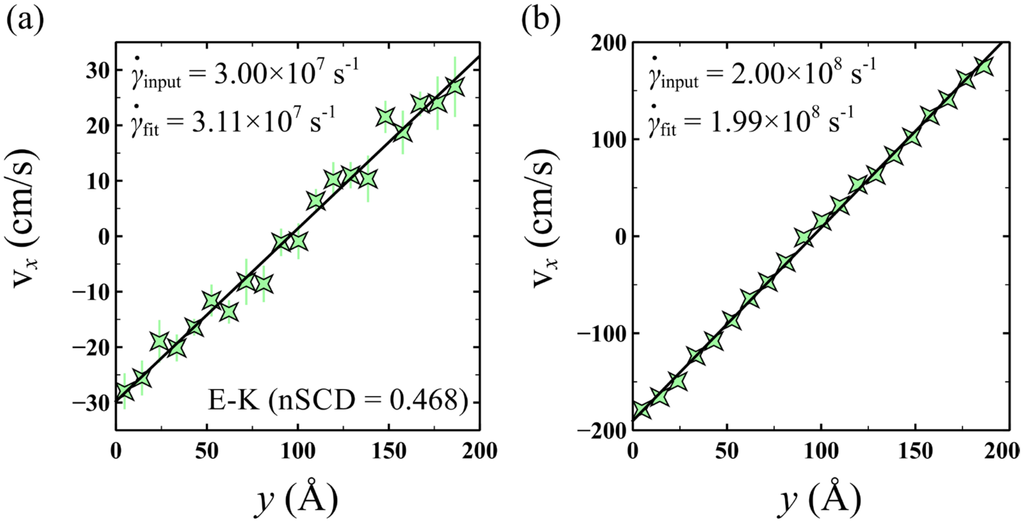

Supplementary Fig. 18. Velocity v_x of the residues in the flow direction x as a function of gradient direction y in the dense phase of a select E−K sequence with $\mathrm{nSCD} = 0.468$ at (a) low and (b) high shear rates used in this work. The solid line corresponds to a linear fit to the data, the slope of which gives the shear rate $\dot{\gamma}_{\text{fit}}$. The value of $\dot{\gamma}_{\text{fit}}$ is nearly identical to the imposed shear rate $\dot{\gamma}_{\text{input}}$ on the dense phase of the condensate. Error bars in all panels indicate standard errors about the mean. The mean values are obtained as an average over $n = 2$ independent simulations, with each replica divided into 3 independent blocks to estimate error bars. Source data are provided as a Source Data file.

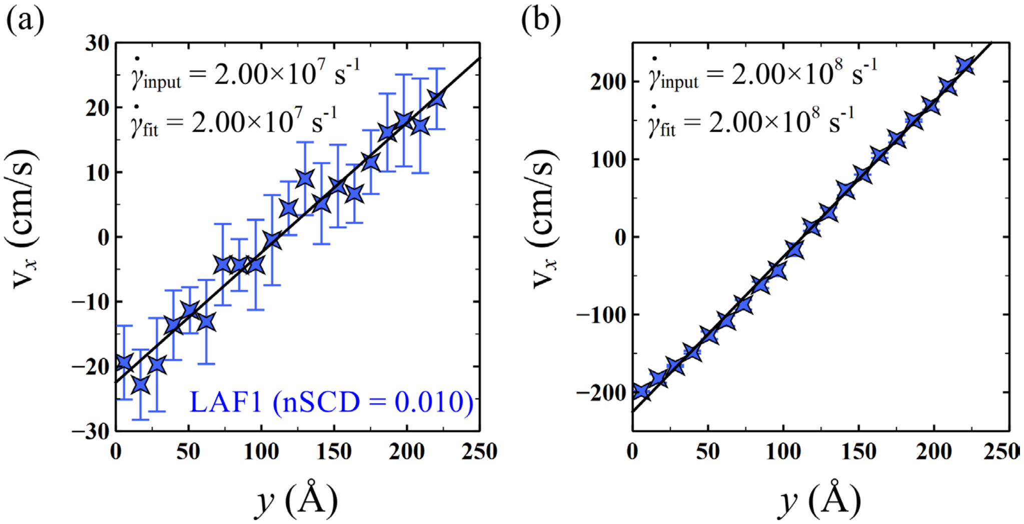

Supplementary Fig. 19. Velocity v_x of the residues in the flow direction x as a function of gradient direction y in the dense phase of a select LAF1 sequence with nSCD $= 0.010$ at (a) low and (b) high shear rates used in this work. The solid line corresponds to a linear fit to the data, the slope of which gives the shear rate $\dot{\gamma}_{\text{fit}}$. The value of $\dot{\gamma}_{\text{fit}}$ is nearly identical to the imposed shear rate $\dot{\gamma}_{\text{input}}$ on the dense phase of the condensate. Error bars in all panels indicate standard errors about the mean. The mean values are obtained as an average over $n = 2$ independent simulations, with each replica divided into 3 independent blocks to estimate error bars. Source data are provided as a Source Data file.

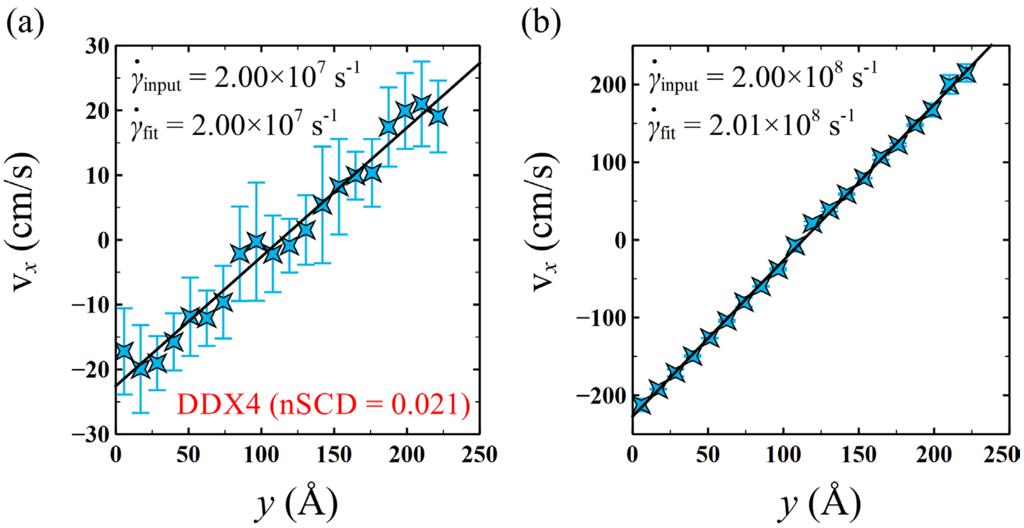

Supplementary Fig. 20. Velocity v_x of the residues in the flow direction x as a function of gradient direction y in the dense phase of a select DDX4 sequence with nSCD $= 0.021$ at (a) low and (b) high shear rates used in this work. The solid line corresponds to a linear fit to the data, the slope of which gives the shear rate $\dot{\gamma}_{\rm fit}$. The value of $\dot{\gamma}_{\rm fit}$ is nearly identical to the imposed shear rate $\dot{\gamma}_{\rm input}$ on the dense phase of the condensate. Error bars in all panels indicate standard errors about the mean. The mean values are obtained as an average over $n = 2$ independent simulations, with each replica divided into 3 independent blocks to estimate error bars. Source data are provided as a Source Data file.

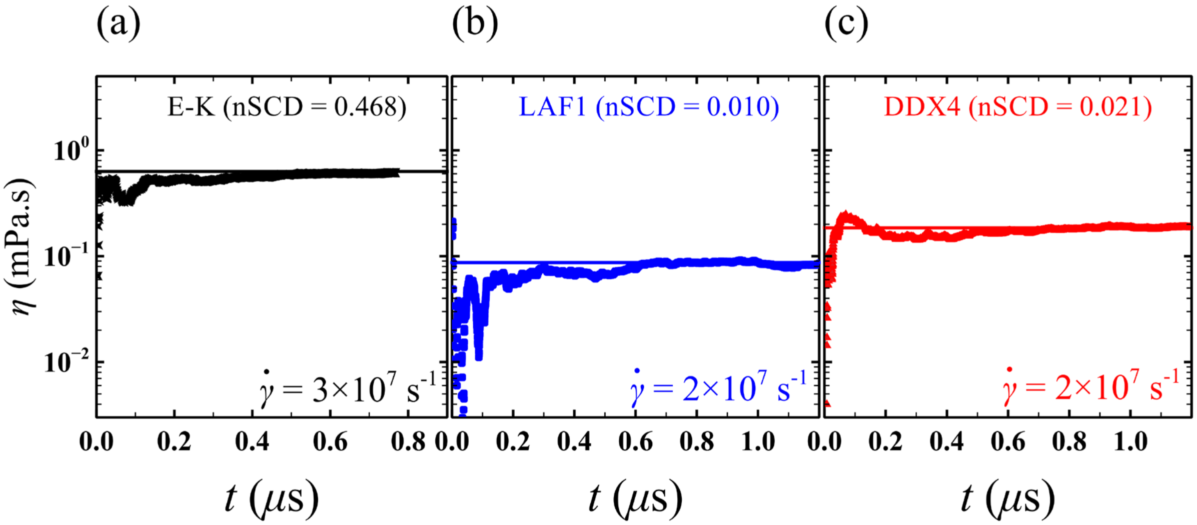

Supplementary Fig. 21. Running average of shear viscosity η as a function of simulation time t at a low shear rate for select (a) E−K (nSCD = 0.468), (b) LAF1 (nSCD = 0.010), and (c) DDX4 (nSCD = 0.021) sequences. The solid lines represent the mean value of η at the low shear rate for the respective sequences. Source data are provided as a Source Data file.

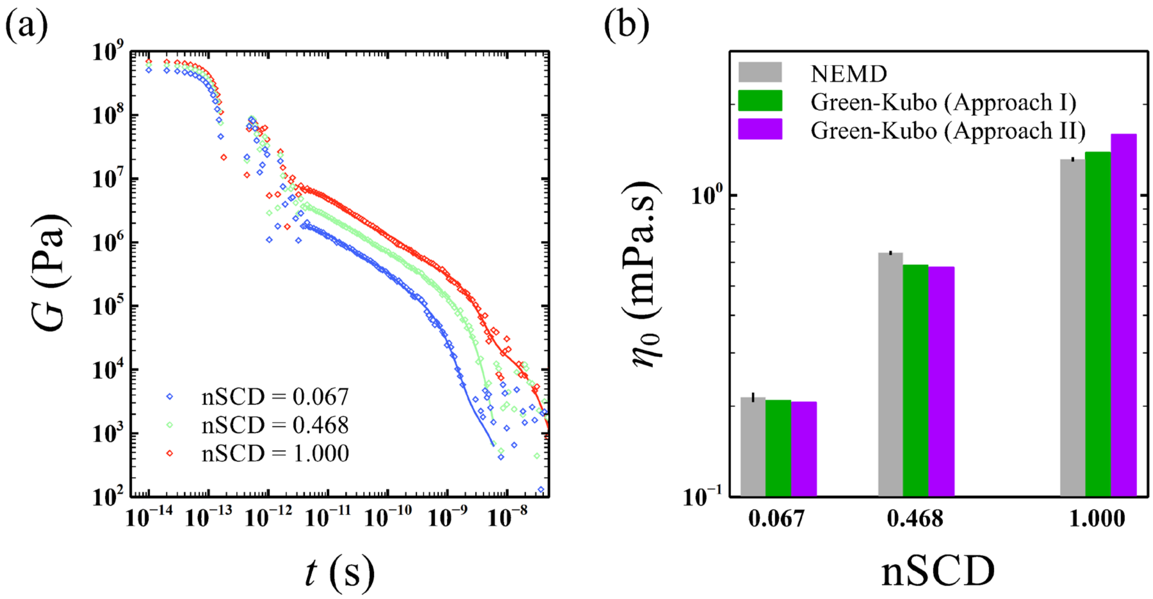

Supplementary Fig. 22. (a) Shear stress relaxation modulus $G(t)$ for select E−K sequences. The solid lines at long time scales represent the fit to a series of Maxwell modes. (b) Comparison between zero-shear viscosity η_0 for the E−K sequences obtained from the NEMD simulations and the Green-Kubo relation. In approach I, η_0 was computed based on the Green-Kubo relation by simply integrating $G(t)$. In approach II, η_0 was computed as per the procedure outlined by Tejedor *et al.*[4] Error bars for η_0 from the NEMD simulations indicate standard errors about the mean. The mean values are obtained as an average over $n = 2$ independent simulations, with each replica divided into 3 independent blocks to estimate error bars. Source data are provided as a Source Data file.

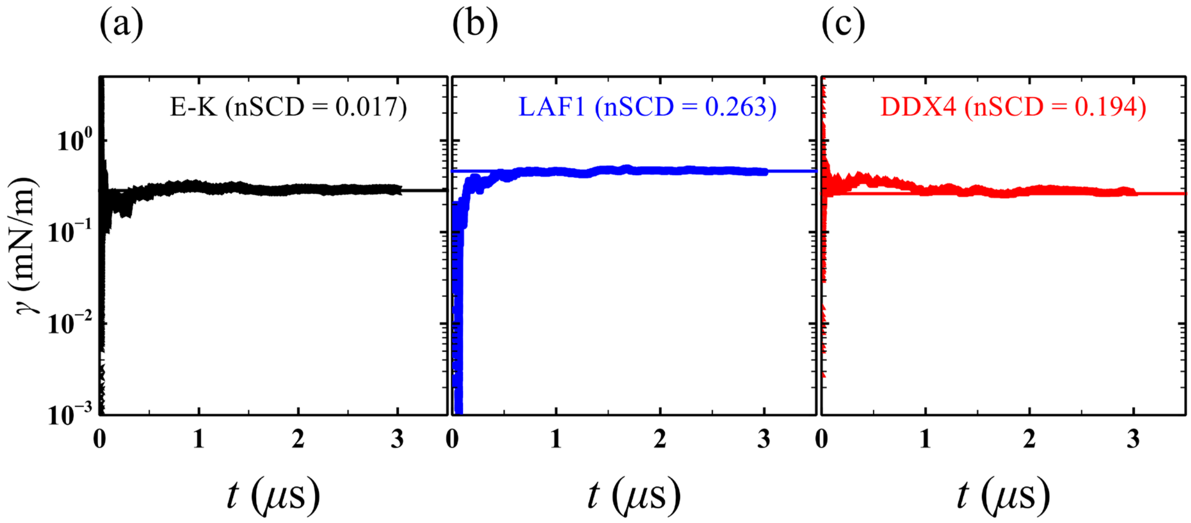

Supplementary Fig. 23. Running average of surface tension γ as a function of simulation time t for select (a) E−K (nSCD = 0.017), (b) LAF1 (nSCD = 0.263), and (c) DDX4 (nSCD = 0.194) sequences. The solid lines represent the mean value of γ for the respective sequences. Source data are provided as a Source Data file.

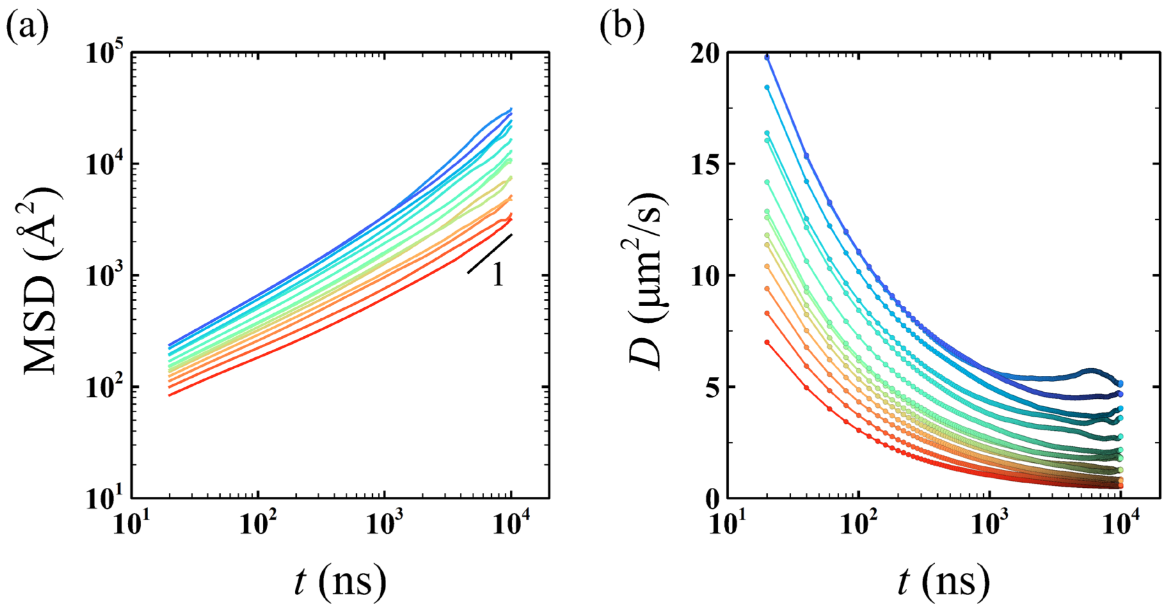

Supplementary Fig. 24. (a) Mean square displacement MSD(t) of the residues of a chain for the E−K sequences simulated using the Martini model. The slope value 1 indicates the diffusive regime. (b) Diffusion coefficient D is extracted from the plateau region observed at long times ($t \geq 5$ μs) where the relation MSD $= 6Dt$ holds. The line color, ranging from blue to red, indicates increasing nSCD. Source data are provided as a Source Data file.

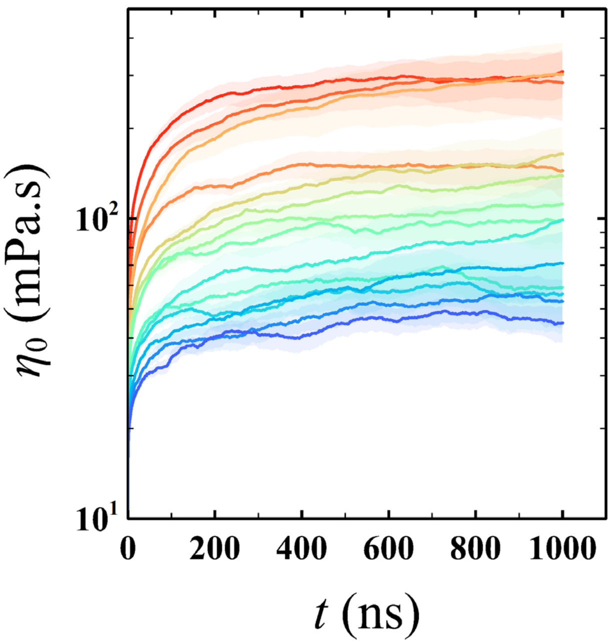

Supplementary Fig. 25. (a) Zero-shear viscosity η_0 is obtained from the plateau region of the Green-Kubo integral observed at long times ($t \geq 0.5\ \mu s$) for the E−K sequences simulated using the Martini model. The line color, ranging from blue to red, indicates increasing nSCD. The shaded color indicate standard errors about the mean for each E−K sequence. The mean values are obtained as an average over $n = 10$ independent simulations, with each replica divided into 2 independent blocks to estimate error bars. Source data are provided as a Source Data file.

Disordered sequence	D (μm^2/s) from experiments*	D (μm^2/s) from simulations#
LAF1 wild-type	0.025 ± 0.009	0.052 ± 0.002
DDX4 wild-type	0.400 ± 0.100	0.061 ± 0.003

Supplementary Table 2. Comparison of dense-phase diffusion coefficients D between simulations (obtained based on the timescales mapped at the single-chain level at infinite dilution) and FRAP experiments for the LAF1 and DDX4 wild-type sequences. In FRAP, the recovery time of the fluorescently labeled protein molecules upon bleaching is measured to estimate the translational diffusion within the condensates. *The experimental conditions pertaining to the D values for LAF1 and DDX4 were $T = 289.15$ K to 291.15 K and $T = 293.15$ K, both in 150 mM NaCl buffer. #The simulation conditions pertaining to the D values for LAF1 and DDX4 were $T = 280$ K and $T = 300$ K with Debye screening length $\ell = 10$ Å, which corresponds to 100 mM salt concentration.

Protein sequence	D_{MD} based on intrinsic MD timescale (m^2/s)	D_{sim} in simulation units ($\sqrt{\varepsilon\sigma^2/m}$ or σ^2/τ)	D_{exp} (m^2/s)	New timescale τ (s) from $D_{\text{sim}} = D_{\text{exp}}$
E−K	3.897×10^{-7}	27.99	1.571×10^{-10}	1.782×10^{-9}
LAF1	1.385×10^{-7}	9.07	0.539×10^{-10}	1.683×10^{-9}
DDX4	0.982×10^{-7}	6.45	0.867×10^{-10}	0.744×10^{-9}

Supplementary Table 3. We first computed the diffusion coefficient D_{MD} of a single-chain based on the intrinsic MD timescale ($\tau = 48.89$ fs) for the E−K, LAF1, and DDX4 sequences (second column). Note that $T = 300$ K was used for the E−K and DDX4 sequences, while $T = 280$ K was used for the LAF1 sequences. Next, we represented the diffusion coefficient in simulation units D_{sim} (third column) by dividing D_{MD} by $\sqrt{\varepsilon\sigma^2/m}$ (or σ^2/τ) for the E−K, LAF1, and DDX4 sequences, respectively. Average mass m of each sequence and length scale $\sigma = 10^{-10}$ m were used in computing the values of $\sqrt{\varepsilon\sigma^2/m}$. We also calculated the experimentally expected diffusion coefficient D_{exp} of a single-chain in water as per the Stokes-Einstein relation (fourth column). Finally, we matched D_{sim} with D_{exp} to obtain new timescales τ (fifth column) that would yield the same single-chain diffusion coefficient in both simulations and experiments for the E−K, LAF1, and DDX4 sequences.

Protein condensate	Protein molecular structure	Viscosity (Pa.s)	Surface tension (mN/m)	Brief method details to get surface tension
LAF1 RGG[3]	Fully disordered	1.62 ± 0.18 Micropipette aspiration	0.159 ± 0.010 Micropipette aspiration	From the intercept of the measured shear rate at different aspiration pressure
polyK[5]	Fully disordered	0.204 SPT	0.017 Fusion	Independent measure of viscosity is used in the surface tension to viscosity ratio obtained based on condensates fusion timescale
polyR[5]	Fully disordered	14.4 SPT	0.1 Fusion	Same as polyK
$[RGRGG]_5$ + dT40[7]	Fully disordered	3 SPT	0.8 Fusion	Same as polyK
polyK (pK) + heparin (H)[1]	Fully disordered	0.30 ± 0.03 Oscillatory microrheology	0.0571 ± 0.0038 Dual optical traps	Dual optical traps to stretch the condensates and the resulting static spring constant is proportional to surface tension
Proline-rich motif (P) + heparin (H)[1]	Fully disordered	0.53 ± 0.04 Oscillatory microrheology	0.067 ± 0.0085 Dual optical traps	Same as pK + H
PGL3[8]	Disordered + folded	1 Dual optical traps	$\sim 4.5 \times 10^{-3}$ Dual optical traps	Dual optical traps to periodically stretch the condensates. Dynamic spring constant and complex shear modulus gives surface tension
FUS[9]	Disordered + folded	0.7 Dual optical traps	$\sim 3.1 \times 10^{-3}$ Dual optical traps	Same as PGL3
SH3 domain (S) + proline-rich motif (P)[1]	S: disordered + folded<br><br>P: fully disordered	3.75 ± 0.14 Oscillatory microrheology	0.0734 ± 0.0058 Dual optical traps	Same as pK + H

Protein condensate	Protein molecular structure	Viscosity (Pa.s)	Surface tension (mN/m)	Brief method details to get surface tension
SH3 domain (S) + lysozyme (L)[1]	S: disordered + folded<br><br>P: fully disordered	10.1 ± 1.1<br>Oscillatory microrheology	0.106 ± 0.015<br>Dual optical traps	Same as pK + H
NPM1[6]	Disordered + folded	0.74<br>SPT	8×10^{-4}<br>Fusion and sessile drop	Fusion: Same as polyK<br><br>Sessile drop: Condensates of various sizes were imaged through prism. Density, length scales from droplet shape, and gravity were used to obtain surface tension

Supplementary Table 4. Surface tension and viscosity of protein condensates and the experimental methods used to measure them in the literature. SPT corresponds to single particle tracking in which the mean square displacement of the fluorescent tracer beads embedded in the condensates is related to the condensate viscosity through the Stokes-Einstein relation.

Protein condensate[1]	Fusion time (ms)	Viscosity (Pa.s)	Surface tension (mN/m)	Surface tension from viscocapillary model (mN/m)
polyK (pK) + heparin (H)	30.3 ± 0.9	0.30 ± 0.03	0.0571 ± 0.0038	0.0585
Proline-rich motif (P) + heparin (H)	39.3 ± 3.9	0.53 ± 0.04	0.067 ± 0.0085	0.0797
SH3 domain (S) + proline-rich motif (P)	384 ± 31	3.75 ± 0.14	0.0734 ± 0.0058	0.0577
SH3 domain (S) + lysozyme (L)	1774 ± 128	10.1 ± 1.1	0.106 ± 0.015	0.0337

Supplementary Table 5. Comparison of estimated surface tension based on the viscocapillary model with the directly measured values for four protein condensates formed by oppositely charged binary mixtures.

References

(1) Ghosh, A.; Kota, D.; Zhou, H.-X. Shear relaxation governs fusion dynamics of biomolecular condensates. *Nature Communications* **2021**, *12* (1), 5995.

(2) Das, R. K.; Pappu, R. V. Conformations of intrinsically disordered proteins are influenced by linear sequence distributions of oppositely charged residues. *Proceedings of the National Academy of Sciences* **2013**, *110* (33), 13392-13397.

(3) Wang, H.; Kelley, F. M.; Milovanovic, D.; Schuster, B. S.; Shi, Z. Surface tension and viscosity of protein condensates quantified by micropipette aspiration. *Biophysical Reports* **2021**, *1* (1), 100011.

(4) Tejedor, A. R.; Collepardo-Guevara, R.; Ramírez, J.; Espinosa, J. R. Time-Dependent Material Properties of Aging Biomolecular Condensates from Different Viscoelasticity Measurements in Molecular Dynamics Simulations. *The Journal of Physical Chemistry B* **2023**, *127* (20), 4441-4459.

(5) Fisher, R. S.; Elbaum-Garfinkle, S. Tunable multiphase dynamics of arginine and lysine liquid condensates. *Nature Communications* **2020**, *11* (1), 4628.

(6) Feric, M.; Vaidya, N.; Harmon, T. S.; Mitrea, D. M.; Zhu, L.; Richardson, T. M.; Kriwacki, R. W.; Pappu, R. V.; Brangwynne, C. P. Coexisting Liquid Phases Underlie Nucleolar Subcompartments. *Cell* **2016**, *165* (7), 1686-1697.

(7) Alshareedah, I.; Thurston, G. M.; Banerjee, P. R. Quantifying viscosity and surface tension of multicomponent protein-nucleic acid condensates. *Biophysical Journal* **2021**, *120* (7), 1161-1169.

(8) Jawerth, L. M.; Ijavi, M.; Ruer, M.; Saha, S.; Jahnel, M.; Hyman, A. A.; Jülicher, F.; Fischer-Friedrich, E. Salt-Dependent Rheology and Surface Tension of Protein Condensates Using Optical Traps. *Physical Review Letters* **2018**, *121* (25), 258101.

(9) Jawerth, L.; Fischer-Friedrich, E.; Saha, S.; Wang, J.; Franzmann, T.; Zhang, X.; Sachweh, J.; Ruer, M.; Ijavi, M.; Saha, S.; et al. Protein condensates as aging Maxwell fluids. *Science* **2020**, *370* (6522), 1317-1323.
