# Supplementary material for: Sequence-dependent material properties of biomolecular condensates and their relation to dilute phase conformations
Source: Nat Commun. 2024 Mar 1;15:1912. doi: 10.1038/s41467-024-46223-w (PMC10907393; doi:10.1038/s41467-024-46223-w)
Supplement: Supplementary file 2 — Reporting Summary [file 41467_2024_46223_MOESM2_ESM.pdf]

nature portfolio

Corresponding author(s): Jeetain Mittal & Dinesh Sundaravadivelu Devarajan

Last updated by author(s): February 6, 2024

Reporting Summary

Nature Portfolio wishes to improve the reproducibility of the work that we publish. This form provides structure for consistency and transparency in reporting. For further information on Nature Portfolio policies, see our Editorial Policies and the Editorial Policy Checklist.

Statistics

For all statistical analyses, confirm that the following items are present in the figure legend, table legend, main text, or Methods section.

n/a	Confirmed	
☐	☒	The exact sample size (*n*) for each experimental group/condition, given as a discrete number and unit of measurement
☐	☒	A statement on whether measurements were taken from distinct samples or whether the same sample was measured repeatedly
☒	☐	The statistical test(s) used AND whether they are one- or two-sided<br>*Only common tests should be described solely by name; describe more complex techniques in the Methods section.*
☒	☐	A description of all covariates tested
☒	☐	A description of any assumptions or corrections, such as tests of normality and adjustment for multiple comparisons
☐	☒	A full description of the statistical parameters including central tendency (e.g. means) or other basic estimates (e.g. regression coefficient) AND variation (e.g. standard deviation) or associated estimates of uncertainty (e.g. confidence intervals)
☒	☐	For null hypothesis testing, the test statistic (e.g. *F*, *t*, *r*) with confidence intervals, effect sizes, degrees of freedom and *P* value noted<br>*Give P values as exact values whenever suitable.*
☒	☐	For Bayesian analysis, information on the choice of priors and Markov chain Monte Carlo settings
☒	☐	For hierarchical and complex designs, identification of the appropriate level for tests and full reporting of outcomes
☐	☒	Estimates of effect sizes (e.g. Cohen's *d*, Pearson's *r*), indicating how they were calculated

Our web collection on statistics for biologists contains articles on many of the points above.

Software and code

Policy information about availability of computer code

Data collection	All coarse-grained simulations reported in this study were performed using HOOMD-blue (version 2.9.3), azplugins (version 0.10.1), LAMMPS (29 October 2020 version), and GROMACS (version 2023.1) simulation software packages.
Data analysis	Mean-squared displacement was computed using the in-house C++ code which reads the trajectories from the simulation package. Viscosity and surface tension were computed directly from the shear stress and pressure tensor information obtained from simulation packages. Radial distribution function was computed using the MDAnalysis suite (version 2.0.0). Contact lifetime was computed using the in-house python code (version 3.9.12) utilizing the trajectory analysis suite MDAnalysis (version 2.0.0).<br><br>Analysis codes and the functionalities within the the software engines used for computing the physical quantities presented in this work are available via the GitHub repository https://github.com/dsd993/CondensateMaterialProperties_Simulations_Analysis.

For manuscripts utilizing custom algorithms or software that are central to the research but not yet described in published literature, software must be made available to editors and reviewers. We strongly encourage code deposition in a community repository (e.g. GitHub). See the Nature Portfolio guidelines for submitting code & software for further information.

Data

Policy information about availability of data

All manuscripts must include a data availability statement. This statement should provide the following information, where applicable:

- Accession codes, unique identifiers, or web links for publicly available datasets
- A description of any restrictions on data availability
- For clinical datasets or third party data, please ensure that the statement adheres to our policy

Source data are provided with this paper. Raw simulation trajectory data used for the analysis to obtain the source data are available from the corresponding authors upon reasonable request. The starting and ending configurations of the different types of simulations performed using the hydropathy scale and Martini models along with the input scripts to initiate the simulations using the publically accessible software engines are available via the GitHub repository https://github.com/dsd993/CondensateMaterialProperties_Simulations_Analysis.

Research involving human participants, their data, or biological material

Policy information about studies with human participants or human data. See also policy information about sex, gender (identity/presentation), and sexual orientation and race, ethnicity and racism.

Reporting on sex and gender	n/a
Reporting on race, ethnicity, or other socially relevant groupings	n/a
Population characteristics	n/a
Recruitment	n/a
Ethics oversight	n/a

Note that full information on the approval of the study protocol must also be provided in the manuscript.

Field-specific reporting

Please select the one below that is the best fit for your research. If you are not sure, read the appropriate sections before making your selection.

[x] Life sciences [ ] Behavioural & social sciences [ ] Ecological, evolutionary & environmental sciences

For a reference copy of the document with all sections, see nature.com/documents/nr-reporting-summary-flat.pdf

Life sciences study design

All studies must disclose on these points even when the disclosure is negative.

Sample size	For the HPS model, we performed n = 2 independent simulations. For the Martini model, we performed 5-10 independent simulations depending on the physical property of interest. The details of these and the exact number of replicas used are given in the Figure captions and in the Methods section.<br><br>These sample sizes were deemed sufficient based on the convergence of the different physical properties over the simulation duration, the results of which are described in detail in the Methods section and in the Supplementary Figures.
Data exclusions	Data were not excluded from any of the analysis/results reported in this study.
Replication	This work is based on two different types of coarse-grained simulations. (1) The simulations using the HPS model were run very long (~1-3 microseconds) and the trajectories of 2 independent replicas were divided into five blocks each for getting the average of all quantities. Error bars on averaged quantities were estimated by computing the standard error of the mean between blocks. The results were consistent irrespective of the number of blocks chosen. (2) For the simulations using the Martini model, the simulations were run long again (~10-20 microseconds) and the physical quantities were computed based on 5 to 10 independent replicas. All attempts at replication were successful. The exact number of independent replicas used depended on the property of interest and has been mentioned in the Methods section and Figure captions of the manuscript.
Randomization	Independent simulations were done with random initial configurations (random starting seeds).
Blinding	The computational analysis performed in this work are objective and hence, blinding was not required.

Reporting for specific materials, systems and methods

We require information from authors about some types of materials, experimental systems and methods used in many studies. Here, indicate whether each material, system or method listed is relevant to your study. If you are not sure if a list item applies to your research, read the appropriate section before selecting a response.

Materials & experimental systems

n/a	Involved in the study
☒	☐ Antibodies
☒	☐ Eukaryotic cell lines
☒	☐ Palaeontology and archaeology
☒	☐ Animals and other organisms
☒	☐ Clinical data
☒	☐ Dual use research of concern
☒	☐ Plants

Methods

n/a	Involved in the study
☒	☐ ChIP-seq
☒	☐ Flow cytometry
☒	☐ MRI-based neuroimaging
